# Forgetting is comparable between healthy young and old people

**DOI:** 10.1038/s41598-024-82570-w

**Published:** 2024-12-28

**Authors:** Martina Studer, Dörthe Heinemann, Klemens Gutbrod, Katharina Henke

**Affiliations:** 1 Department of Pediatric Neurology and Developmental Medicine, University Children’s Hospital Basel (UKBB), Basel, Switzerland; 2 Department of Clinical Research, University of Basel, Basel, Switzerland; 3 Department of Neurology, Inselspital, Bern University Hospital, University of Bern, Bern, Switzerland; 4 Neurozentrum Bern, Bern, Switzerland; 5 Institute of Psychology, University of Bern, Bern, Switzerland; 6 Department of Psychology, University of Basel, Basel, Switzerland

**Keywords:** Episodic memory, Verbal recall, Verbal recognition, Forgetting over one week, Executive functions, Subjective sleep quality, Healthy adults, Psychology, Neurology

## Abstract

Aging is typically associated with declines in episodic memory, executive functions, and sleep quality. Therefore, the sleep-dependent stabilization of episodic memory is suspected to decline during aging. This might reflect in accelerated long-term forgetting, which refers to normal learning and retention over hours, yet an abnormal retention over nights and days. Accelerated long-term forgetting has been observed in dementia, mild cognitive impairment, and in people with memory complaints. Here, we explored whether accelerated long-term forgetting also manifests in healthy aging. We investigated verbal episodic memory in 236 healthy men and women between 18 and 77 years of age. All participants were mentally intact in terms of executive functions, working memory, episodic memory, verbal intelligence, and mood. We related their forgetting rates over one week following learning to their subjective sleep quality and executive functions. Fifteen words were freely recalled and then recognized among 30 distractor words at 30 min and again at one week following learning. Although the healthy older adults compared to the healthy younger adults reported a diminished sleep efficiency and learned fewer words, they exhibited no disproportionate forgetting over days. Hence, accelerated long-term forgetting is not present in healthy aging but might be a first sign of memory dysfunction due to neuropathology.

**Supplementary Information:**

The online version contains supplementary material available at 10.1038/s41598-024-82570-w.

## Introduction

A concerning issue of our aging society is cognitive decline, particularly the decline of episodic memory^1,2^. Episodic memory stores information about personal events and critically depends on the hippocampus, a bilateral brain structure in the medial temporal lobe^3^. The neurobiological processes underlying episodic memory impairment in normal aging are distinct from those underlying pathological aging such as Alzheimer’s Disease^4^. Therefore, an easy way to distinguish between pathological aging and normal aging on an individual basis is much sought. Here, we suggest that the assessment of delayed memory recall might be a way to distinguish between pathological and healthy aging^5^ because accelerated long-term forgetting has been identified in several neurological conditions but we find no evidence for accelerated long-term forgetting in a large sample of healthy adults.

Accelerated long-term forgetting is much underdiagnosed because its assessment is not included in standard neurological and neuropsychological check-ups. Accelerated long-term forgetting is a memory deficit that features a normal learning and normal retention of newly experienced episodes over hours, followed by increased forgetting over subsequent days and weeks. To uncover accelerated long-term forgetting, patients must be asked to retrieve the learned information several days following learning. This means that clinicians assess patients on at least two separate occasions. The cause for accelerated long-term forgetting might be subtle learning deficits^6^ or an impaired delayed memory consolidation during sleep or wakefulness^7–9^. The process of memory consolidation stabilizes a memory after its inception through hippocampal – neocortical interactions^10^. Although diminished slow-wave sleep has been observed and related to reduced episodic memory in older adults^11^, accelerated long-term forgetting seems not to be caused by a disruption of sleep-dependent memory consolidation^12^. Conversely, the here reported healthy older adults exhibited diminished sleep efficiency but no accelerated long-term forgetting.

Accelerated long-term forgetting has been found in epilepsy^13^, traumatic brain injury^14,15^, transient ischemic attacks, stroke^16,17^, pre-clinical dementia^18,19^, amnestic mild cognitive impairment^20,21^, and even in healthy older individuals with memory complaints but no diagnosis of mild cognitive impairment^21,22^. Evidence suggests that patients with memory disorders exhibit both a learning deficit and rapid forgetting as indicated by their free recall performance. However, when learning performance is statistically controlled between patients and healthy controls, the rate of forgetting in terms of recognition memory is comparable between patients and healthy controls^23–25^. This suggested that faster forgetting in patients may at least partly originate in a learning deficit. Since the publication of these early findings, a debate is ongoing whether accelerated long-term forgetting is associated with a learning or a consolidation deficit and whether accelerated long-term forgetting is also associated with healthy ageing or whether it signals neuropathology^6^.

The current evidence is inconclusive regarding the question whether accelerated long-term forgetting afflicts healthy older adults or not. However, current evidence is conclusively showing that age has a negative impact on learning performance^26–29^. Regarding forgetting, Rivera-Lares and colleagues found similar forgetting rates over 24 h in healthy young and older adults using a cued recall measure of memory, which assesses episodic memory^29^. Because aging and dementia concern episodic memory rather than other types of memory, a process-pure retrieval measure, such as cued or free recall, is essential in the assessment of aging-associated deficits in memory. Hulicka and Weiss reported similar forgetting rates even over a whole week in healthy young and older adults (hospitalized male veterans) using a cued recall measure of memory^26^.When the memory measure was recognition performance, which simultaneously assesses episodic memory, familiarity and priming and hence profits from various types of memory and memory systems^30^, older versus young age is neither differentially affected by long-term forgetting as measured over 48 h^31^. Even for a study-test interval of 27 weeks and even for the comparison of people with subjective memory complaints with young, healthy people, Spikman and colleagues did not find differential rates of forgetting in terms of recognition performance^32^. On the other hand, Hupert and Kopelman reported accelerated long-term forgetting in older versus young people in terms of recognition performance over one week^27^. Hence, memory retrieval in terms of recognition performance yielded contradictory results. But also with the recall memory measure and diverse study-test intervals, several authors reported accelerated long-term forgetting in older versus young healthy people (study-test interval of 55 min^33^; study-test interval of 24 h^28^; study-test interval of one week^28,34,35^). Interestingly, in the study of McGibbon and colleagues^33^ those older participants, who learned more slowly and forgot more rapidly, reported more subjective memory complaints versus those older participants, who learned rapidly. Because McGibbon’s participants in the older age group were not screened for cognitive health, the slow learning in certain participants might reflect preclinical neuropathology. It should be noted that the studies, which found accelerated long-term forgetting in older versus young people, did not screen their participants for cognitive dysfunction. They might therefore have included older people with preclinical neuropathology, which might have caused the group effect of accelerated long-term forgetting^27,28,34,35^. To conclude, studies obtained mixed results regarding accelerated long-term forgetting in healthy aging. This inconclusiveness might result from (1) the application of memory tests that assess either episodic memory (recall) or several memory types (recognition) including memory types that can compensate for a poor episodic memory, (2) the use of study-test delays that range from an hour to 27 weeks, (3) the inclusion of cognitively impaired older people, (4) restricting recruitment to people younger than 59 years of age and (5) small sample sizes. In the current study, we explored whether healthy older people would exhibit accelerated long-term forgetting compared to younger people, when the above mentioned issues are fixed by (1) including both a recognition and a recall measure for completeness, (2) using the standard study-test interval of one week for the assessment of accelerated long-term forgetting, (3) extensively screening both younger and older participants regarding their cognitive and mental health, (4) including people from 18 to 77 years of age, and (5) using larger sample sizes.

We examined accelerated long-term forgetting in healthy 236 men and women between 18 and 77 years of age. These participants were assigned to three age groups: young adults (18–29 years, *n* = 88), middle aged adults (30–59 years, *n* = 103), and older adults (60–77 years, *n* = 45). Due to the ongoing Covid pandemic, the recruitment of older participants was particularly difficult. All participants were mentally healthy in terms of executive functions, working memory, episodic memory, verbal intelligence, and mood. We related their forgetting rates over one week following learning to their subjective sleep quality and executive functions because both sleep and executive functions may deteriorate in the aging process and may affect memory performance^11,36^. Verbal learning and retrieval performance was examined using a Swiss adaptation of the Rey Auditory Verbal Learning Test (Auditory-Verbal Learning Test, AVLT)^37^. In this test, participants learn fifteen unrelated nouns in five learning runs. Each learning run was followed by an immediate free recall without corrective feedback. We defined verbal learning performance as the sum of all recalled words across the five learning runs. A distractor word list of 15 new nouns was then learned in a single learning run and immediately recalled. Following this interference, participants freely recalled again the nouns from the initial learning list (2-minute recall) and again at 30 min (30-minute recall) and again at one week (1-week recall) following learning. Both the 30-minute recall and the 1-week recall were followed by a recognition test, which comprised the same 45 words at both time points. The recognition list included the 15 nouns from the initial learning list, the 15 nouns from the distractor list, and 15 new nouns that had not been presented before. Neither the distractor words nor the new words were semantically or phonologically related to the learned words from the initial list.

## Results

### Demographics, gender, and subjective sleep quality

Age groups did not differ regarding gender, years of education, depression scores and subjective sleep quality (Table 1). However, multiple-choice vocabulary performance, an approximation of verbal IQ^38^, was worse in the young age group versus the middle age group and the old age group. Because the verbal IQ and multiple-choice vocabulary performance do influence learning and memory performance^39^, we included multiple-choice vocabulary performance as a covariate in the computed analyses of variance. The older adults in our sample also learned fewer words than the younger adults (Table 2). Therefore, the number of learned words was included as a second covariate in the analyses of variance computed on the retrieval performance.

Women learned more words across the five learning runs than men (Table 2) with young women excelling. However, men and women did not differ in their recall or recognition performance at any time point, and gender did not interact with age group. Therefore, we only report age-specific recall and recognition performance in Table 2.

Total PSQI Sleep Score did not differentiate between age groups (Table 1). Neither did the PSQI Subscales Sleep Quality, Sleep Latency, Sleep Duration and Sleep Medication differentiate between age groups. However, the young age group reported (1) a better sleep efficiency (percentage of time spent asleep while in bed) than the old age group, (2) more Daytime Sleep than the old age group, and (3) fewer sleep disturbances than the middle age group. There were no interactions between gender and age groups on any of the PSQI Subscales. Measured over all age groups, 73% of participants rated their sleep as good, 24% rated their sleep as poor; and 3% had a PSQI Total Score over 10 indicating a chronic sleep problem. Good and poor sleepers were evenly distributed across the age groups.

**Table 1:** Demographics and PSQI outcome across age groups.

	Age groups			Test statistics	*p*-value	Effect size	Post-hoc comparisons* 1) Young age vs. middle age 2) Young age vs. old age 3) Middle age vs. old age 4) Female vs. male
	Young age (18–29 years)	Middle age (30–59 years)	Old age (60 + years)	Test of significance			
Demographics							
Sample size	*n* = 88	*n* = 103	*n* = 45				
Age, *M (SD)*	23.91 (3.10)	47.54 (9.76)	66.07 (3.76)				
Years of education, *M (SD*)	13.85 (2.13)	14.52 (2.88)	14.53 (2.55)	*F* (2,233) = 1.95	0.15		
Sex female, n (%)	42 (48%)	64 (62%)	24 (53%)	*X*^*2*^ (2) = 4.05	0.13		
Depression rating (DESC), *M (SD*)	3.18 (2.84)	2.76 (2.99)	3.15 (3.23)	*F* (2,233) = 0.55	0.58		
Multiple choice vocabulary test (MWT-B), *M(SD)*	111.83 (12.12)	119.94 (14.03)	123.96 (11.99)	*F* (2,233) = 15.79	< 0.001***	*η*_*p*_^*2*^ *=* 0.12	*1) p* < .001, *M* = -8.11, 95% CI [-12.70, -3.53] 2) *p* < .001, *M*= -12.13, 95% CI [-17.86, -6.40] *3) ns*
PSQI – Total Score, M(SD)	4.29 (2.11)	4.65 (2.72)	4.28 (2.47)	*F* (2,230) = 0.64	0.53		
PSQI – Sleep Quality *M(SD)*	0.75 (0.60)	0.90 (0.69)	0.67 (0.61)	*F* (2,230) = 2.44	0.09		
PSQI – Sleep Latency *M(SD)*	1.03 (0.69)	0.94 (0.80)	0.95 (0.87)	*F* (2,230) = 0.364	0.70		
PSQI – Sleep Duration *M(SD)*	0.29 (0.53)	0.38 (0.61)	0.33 (0.57)	*F* (2,230) = 0.605	0.55		
PSQI – Sleep Efficiency *M(SD)*	0.25 (0.69)	0.38 (0.72)	0.63 (0.93)	*F* (2,230) = 3.62	0.028*	*η*_*p*_^*2*^ = 0.03	*1) ns* 2) *p* < .05, *M*= -0.38, 95% CI [-0.71, -0.04] *3) ns*
PSQI – Sleep Disturbances *M(SD)*	0.98 (0.30)	1.17 (0.47)	1.05 (0.38)	*F* (2,230) = 5.933	0.003**	*η*_*p*_^*2*^ = 0.05	*1) p* < .01, *M*= -0.20, 95% CI [-0.25, 0.11] 2) *ns* *3) ns*
PSQI – Sleep Medication *M(SD)*	0.06 (0.35)	0.12 (0.49)	0.05 (0.21)	*F* (2,230) = 0.710	0.49		
PSQI – Sleep Daytime *M(SD)*	0.93 (0.70)	0.76 (0.62)	0.60 (0.58)	*F* (2,230) = 4.021	0.02*	*η*_*p*_^*2*^ = 0.03	*1) ns* 2) *p* < .05, *M* = 0.33, 95% CI [0.04, 0.61] *3) ns*

*M*: mean; *ns*: not significant; *SD*: standard deviation. *DESC*: Rasch basiertes Depressionsscreening. *MWT-B*: Mehrfachwahl-Wortschatz-Intelligenztest. *PSQI*: Pittsburgh Sleep Quality Index. Significance value: *p < .05, **p < .01 ***p < .001.

### Recall performance over one week

**Fig. 1:**
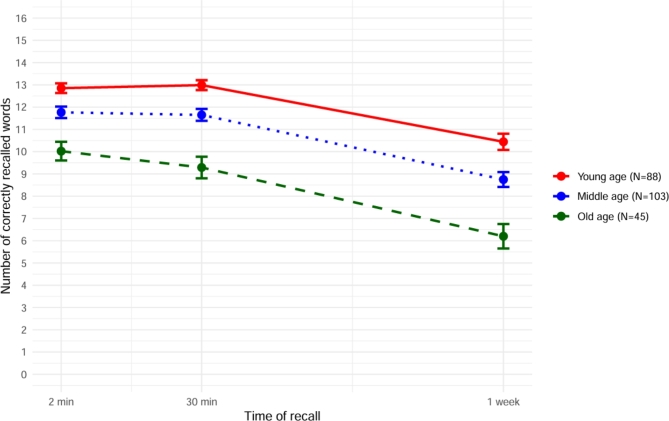
Mean verbal recall performance over time plotted for each age group. Error bars represent estimated standard error of the mean.

A repeated measures ANCOVA with the covariates ‘learning performance’ and ‘multiple choice vocabulary performance’ and the independent variables ‘age group’ and ‘time’ (3-levels: 2-min, 30-min, 1-week) revealed a significant main effect of time (*F*(1.53, 352.69) = 12.90, *p* < .001, *η*_*p*_^*2*^ = 0.05) and main effect of age group (*F*(2,231) = 5.46, *p* < .01, *η*_*p*_^*2*^ = 0.05) on recall performance but – importantly – no significant interaction between time × age group (*F*(3.05, 352.69) = 1.27, *p* = .28). Hence, old age was not associated with a disproportionate memory loss over one week (Fig. 1). Pairwise post-hoc comparisons indicated that all age groups recalled more words at 2 min than 1 week following learning (*p* < .001, *M* = 3.05, 95% CI [2.60, 3.49]) and at 30 min than 1 week following learning (*p* < .001, *M* = 2.84, 95% CI [2.43, 3.24]). Both the young age group (*p* < .01, *M* = 1.23, 95% CI [0.31, 2.15]) and the middle age group (*p* < .05, *M* = 0.92, 95% CI [0.13, 1.72]) recalled more words than the old age group at all time points.

Another ANCOVA with the covariates ‘learning performance’ and ‘multiple choice vocabulary performance’ and the independent variable ‘age group’ was run with the dependent variable ‘recall loss over 1-week’ (30-min recall – 1-week recall) to measure differential long-term forgetting between age groups more directly. This analysis showed no significant main effect of age group on the recall loss from 30 min to 1week following learning (Table 2). Therefore, old age seems not to be associated with a disproportionate episodic memory loss over one week.

**Table 2:** Verbal learning and memory performance (recall and recognition) as well as false alarms and forgetting over time (recall and recognition loss) across age groups.

	Age groups			Test Statistics	*p*-value	Effect size	Post-hoc comparisons*
	Young age (18–29 years) n = 88	Middle age (30 – 59 years) n = 103	Old age (60 + years) n = 45	Test of significance			1) young age vs. middle age 2) young age vs. old age 3) middle age vs. old age
Learning performance							
Learning (sum L1-L5), *M (SD)* ^*1*^	59.76 (6.63)	55.67 (7.13)	50.62 (9.64)	Age: F(2,232) = 30.55	< 0.001***	*η*_*p*_^*2*^ = 0.21	1)* p* < 0.001, *M* = 5.24, 95% CI [3.08, 7.41] 2)* p* < 0.001, *M* = 10.86, 95% CI [8.09, 13.63] 3) *p* < 0.001, *M* = 5.62, 95% CI [3.04, 8.20]
	Fe: 60.31 (6.40) Ma: 59.26 (6.86)	Fe: 57.75 (6.25) Ma: 52.26 (7.24)	Fe: 54.83 (7.55) Ma: 45.81 (9.67)	Sex: F(1,229) = 27.15 Age x sex: F(2,229) = 4.65	< 0.001*** < 0.05*	*η*_*p*_^*2*^ = 0.11 *η*_*p*_^*2*^ = 0.04	Female vs. male: *p* < 0.001, *M* = 5.00, 95% CI [3.11, 6.89]
Recall performance							
2-min recall, *M (SD)*^*2*^	12.85 (2.03)	11.77 (2.61)	10.02 (2.81)	Age: F (2, 231) = 1.78	0.17		
30-min recall, *M (SD)*^*2*^	12.99 (2.08)	11.65 (2.72)	9.29 (3.26)	Age: F (2,231) = 5.79	0.004**	*η*_*p*_^*2*^ = 0.05	1) *ns* 2)* p* < 0.01, *M* = 1.26, 95% CI [0.49, 2.03] 3) *p* < 0.01, *M* = 1.04, 95% CI [0.37, 1.70]
1-week recall, *M (SD)*^*2*^	10.44 (3.44)	8.75 (3.40)	6.20 (3.68)	Age: F (2,231) = 4.22	0.02*	*η*_*p*_^*2*^ = 0.04	1) *ns* 2)* p* < 0.05, *M* = 1.73, 95% CI [0.55, 2.91] 3) *p* < 0.05, *M* = 1.15, 95% CI [0.13, 2.17]
Recognition performance							
30-min recognition (hits), *M (SD)* ^*2*^	14.51 (0.98)	14.18 (1.24)	13.64 (1.57)	Age: F(2,231) = 1.26	0.29		
30-min recognition (Pr), *M (SD)*^*2*^	14.25 (1.39)	13.61 (1.73)	12.13 (2.36)	Age: F(2,231) = 6.45	< 0.01**	*η*_*p*_^*2*^ = 0.05	1)* ns* 2)* p* < 0.001, *M* = 1.13, 95% CI [0.47, 1.78] 3) *p* < 0.01, *M* = 0.92, 95% CI [0.36, 1.48]
1-week recognition (hits), *M (SD)* ^*2*^	13.80 (1.60)	13.21 (1.93)	12.29 (2.63)	Age: F(2,231) = 1.10	0.33		
1-week recognition (Pr), *M (SD)*^*2*^	13.06 (2.49)	11.46 (3.33)	8.87 (4.28)	Age: F(2,231) = 6.94	< 0.01***	*η*_*p*_^*2*^ = 0.06	1) *ns* 2)* p* < 0.001, *M* = 2.28, 95% CI [1.07, 3.50] 3) *p* < 0.01, *M* = 1.53, 95% CI [0.48, 2.57]
False alarms in recognition performance							
30-min false alarms, *M (SD) *^*2*^	0.24 (0.61)	0.57 (0.97)	1.51 (1.83)	Age: F (2,231) = 7.19	< 0.001***	*η*_*p*_^*2*^ = 0.06	1)* ns* 2)* p* < 0.01, *M* = -0.76, 95% CI [-1.29, -0.24] 3) *p* < 0.01, *M* = -0.66, 95% CI [-1.11, -0.20]
1-week false alarms, *M (SD) *^*2*^	0.74 (1.28)	1.76 (2.29)	3.51 (3.53)	Age: F(2,231) = 7.42	< 0.001***	*η*_*p*_^*2*^ = 0.06	1) *ns* 2)* p* < 0.001, *M* = -1.78, 95% CI [-2.90, -0.66] 3) *p* < 0.01, *M* = -1.20, 95% CI [-2.17, -0.24]
Recall and recognition loss over time							
Recall loss over 1 week, *M (SD)* ^*2*^	2.55 (2.34)	2.90 (2.43)	3.09 (2.44)	Age: F(2,231) = 0.56	0.57		
Recognition loss (hits) over 1 week, *M (SD)*^*2*^	0.72 (1.40)	0.97 (1.46)	1.36 (2.10)	Age: F(2,231) = 0.16	0.85		
Recognition loss (Pr) over 1 week, *M (SD)*^*2*^	1.19 (2.08)	2.16 (2.48)	3.27 (3.39)	Age: F(2,231) = 2.42	0.09		

Note. *Fe*: female; *M*: mean; *Ma*: male; *ns*: not significant, *Pr*: Discrimination score (hits – false alarms), *SD*: standard deviation. Significance value: *p < .05, **p < .01 ***p < .001. ^1^controlled for multiple choice vocabulary performance, ^2^controlled for learning and multiple choice vocabulary performance.

### Recognition performance in terms of hits (H) over one week

**Fig. 2:**
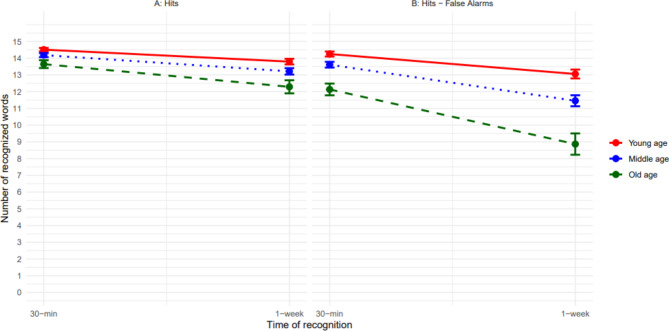
Mean verbal recognition performance over time plotted for each age group. On the left side (Panel A), recognition performance is plotted in terms of hits; on the right side (Panel B) recognition performance is plotted in terms of hits–false-alarms. Error bars represent estimated standard error of the mean.

A repeated measures ANCOVA with the covariates ‘learning performance’ and ‘multiple choice vocabulary performance’ and the independent variables ‘age group’ and ‘time’ (2-levels: 30-min, 1-week) revealed a significant main effect of time on the number of H (*F*(1, 231) = 8.77, *p* < .01, *η*_*p*_^*2*^ = 0.04) and neither a main effect of age group (*F*(2, 231) = 1.50, *p* = .23) nor an interaction between time and age group (*F*(2, 231) = 0.16, *p* = .85). Hence, recognition performance in terms of H dropped over time for all people and did not differ between age groups at any time point (Fig. 2 Panel A). The post-hoc comparison indicated that more old words were recognized correctly at 30 min compared to 1 week following learning (*p* < .001, *M* = 0.97, 95% CI [0.76, 1.19]).

A further ANCOVA with the covariates ‘learning performance’ and ‘multiple choice vocabulary performance’ and the independent variable ‘age group’ was run with the dependent variable ‘recognition loss in terms of H over 1-week’ (30-min H – 1-week H) to measure differential long-term forgetting between age groups more directly. There was no significant effect of age group on recognition loss in terms of H (Table 2). Hence, older adults seem not to experience a disproportionate recognition loss in terms of hits over one week.

### Recognition performance in terms of hits minus false alarms (pr) over one week

A repeated measures ANCOVA with the covariates ‘learning performance’ and ‘multiple choice vocabulary performance’ and the independent variables ‘age group and ‘time’ (2-levels: 30-min, 1-week) and the dependent variable Pr was computed. This ANCOVA revealed a significant main effect of time (*F*(1, 231) = 19.56, *p* < .001, *η*_*p*_^*2*^ = .08) and a significant main effect of age group (*F*(2, 231) = 8.61, *p* < .001, *η*_*p*_^*2*^ = .07) but no interaction between time and age group (*F*(2, 231) = 2.42, *p* = .09, Fig. 2 Panel B). Post-hoc comparisons confirmed that recognition performance Pr dropped from 30 min to 1 week following learning (*p* < .001, *M* = 2.12, 95% CI [1.78, 2.46]) for all participants and that the old age group exhibited a significantly reduced recognition performance for both time points (30 minutes and 1 week following learning) compared to both the young age group (*p* < .001, *M* = -1.71, 95% CI [-2.71, -0.70]) and the middle age group (*p* < .01, *M* = -1.22, 95% CI [-2.09, -0.35]).

A further ANCOVA with the covariates ‘learning performance’ and ‘multiple choice vocabulary performance’ and the independent variable ‘age group’ was run with the dependent variable ‘recognition loss in terms of Pr over 1-week’ (30-min Pr – 1-week Pr). This analysis did not reveal an age effect on recognition loss (Table 2). Note, however, that the *p*-value in this analysis was 0.09 suggesting a trend towards more false alarms in older than younger participants at one week versus 30 min following learning. Also Fig. 2 (Panel B) indicates that the older versus younger participants exhibited a steeper forgetting slope in terms of recognition due to increased false alarm rates.

### More FA during recognition with advancing age

A repeated measures ANCOVA with the covariates ‘learning performance’ and ‘multiple choice vocabulary performance’ and the independent variables ‘age group’ and ‘time’ (2-levels: 30-min, 1-week) and the dependent variable ‘FA’ revealed a significant main effect of time (*F*(1, 231) = 11.22, *p* < .001, *η*_*p*_^*2*^ = 0.05), age group (*F*(2, 231) = 9.42, *p* < .001, *η*_*p*_^*2*^ = 0.08), and a significant interaction between time and age group (*F*(2, 231) = 3.08, *p* = .048, *η*_*p*_^*2*^ = 0.03). Post-hoc comparisons revealed more FA at 1-week compared to 30-min following learning across all age groups (*p* < .001, *M* = 1.18, 95% CI [0.92, 1.44]). Participants in the old age group made significantly more false alarms compared to participants in the young age group (*p* < .001, *M* = -1.27, 95% CI [0.55, 1.99]) and participants in the middle age group (*p* < .01, *M* = 0.93, 95% CI [0.31, 1.55]). Although all age groups exhibited a significant increase in FA over time (young age: *p* < .01, *M* = 0.69, 95% CI [0.25, 1.13]; middle age: *p* < .001, *M* = 1.16, 95% CI [0.78, 1.53]); old age: *p* < .001, *M* = 1.70, 95% CI [1.09, 2.31]), Fig. 2 (Panel B) shows that the significant interaction between time and age group was driven by the old age group that exhibited the largest increase of FA over one week.

We further analyzed whether false alarms concerned rather distractor words or new words in the recognition list. Older participants committed more false alarms to distractor nouns in the recognition list (= nouns from the distractor list that was learned in one learning run) compared to both other age groups (Supplementary Table S1). This was true for both recognition time points. In addition, older participants committed more false alarms to new nouns in the recognition list than participants from the middle age group. Surprisingly, this was only true for the 30-min recognition, i.e., at a time point, when the new nouns were contextually unfamiliar because they were presented for the first time.

### Predictors of the forgetting rate over one week

We used multiple linear regression analysis to find out whether demographic variables (age and years of education), intelligence (the individual score of the multiple choice vocabulary performance), learning performance (sum of remembered words across the five runs), subjective sleep quality (the individual scores of all PSQI subscales), working memory, and executive functions (interference control, verbal fluency) would predict verbal forgetting over one week in the entire group of participants. We computed this multiple linear regression analysis with recall loss over 1-week (30-min recall – 1-week recall) as dependent variable. *R*^*2*^ change was used as an indicator of the individual variance explained by the variables. Recall loss over time was not predicted by any predictor, with an insignificant regression analysis (*F*(14,217) = 0.97; *p* = .48; corrected *R*^*2*^ = 0.00).

## Discussion

Accelerated long-term forgetting was observed in patients with dementia, amnestic mild cognitive impairment and even in undiagnosed people with memory complaints. This begs the question whether the healthy aging trajectory is also affected by accelerated long-term forgetting. Though we find that healthy older adults compared to younger adults report a diminished sleep efficiency and learned not as many words, they exhibited no accelerated long-term forgetting in terms of free recall of nouns and in terms of correctly recognized nouns from the learning list. Therefore, accelerated long-term forgetting might reflect lurking brain pathology and imminent disease.

While older versus younger age was not associated with disproportionate forgetting over a week, older age was associated with a less effective encoding of words as previously observed^26–29^. This slowed encoding protracted to a less productive recall at 2 minutes, 30 minutes and at one week following encoding because the older adults had acquired fewer words to remember. Our older participants’ decrease in episodic encoding is probably not caused by hippocampal dysfunction because this would simultaneously reflect in a diminished long-term consolidation with a poor delayed retrieval. Rather, their decrease in episodic encoding may be caused by reduced processing speed and by declining executive functions^40–42^. Although the older participants exhibited intact executive functions commensurate with age-norms, their executive functions still succumb to the young participants’. Because the aging process may be related to declining executive functions, especially from the age of 55 onwards^42^, our older participants may have used less effective encoding strategies than the younger participants. Another cause for the diminished episodic encoding are the commonly found reductions in the integrity of white matter microstructure in bilateral fronto-temporal pathways and the genu of the corpus callosum in healthy older people^41^. This reduced integrity of white matter microstructure may have reduced the efficiency of encoding in the older participants by disrupting the connections within distributed networks.

We included the differential learning performance in our sample as a covariate in the analyses of variance focused on the retrieval performance because we were interested in assessing hippocampus-dependent long-term memory consolidation independently of the learning performance. Interestingly, even when the covariate ‘learning performance’ was excluded from the ANCOVAs, which were computed with the dependent variables recall loss and recognition loss (in terms of hits) over one week, age remained unassociated with the recall and recognition loss over the week. This finding bolsters the conclusion that healthy aging is not associated with accelerated long-term forgetting. Our results are in line with results from a recent study in which forgetting over 24 h was independent of the initial degree of learning in all age groups^29^. It is remarkable that sleep fragmentation was increased in our older versus the younger participants with no effect on long-term memory consolidation. This lack of effect of intra-sleep awakenings on memory consolidation may result from awakenings between sleep cycles with no disruption of the sleep cycles themselves^43^. It is also possible that memory consolidation was not critically dependent on the quality of sleep because it also happened during wakefulness.

Contrary to other studies on accelerated long-term forgetting in healthy adults^28,34,35^, we examined the mental functions and mood of the included 236 men and women carefully to ensure a normal mental status. It hence appears that in truly healthy younger and older adults hippocampal processing is intact and promotes a normal long-term memory consolidation over a week. Authors reporting accelerated long-term forgetting in healthy older adults^27,28,33–35^ may have obtained this positive result because they included participants with premorbid neuropathology due to an insufficient assessment of mental functions^27,28,33–35^ and due to the recruitment of few participants^33,35^. Other studies on accelerated long-term forgetting in healthy adults had not included older adults at all, i.e., people above 59 years of age^28^.

A large body of evidence indicates that the recognition performance of aging people is characterized by a tendency for false alarms^44–47^ False alarms appear to be related to an increased reliance on familiarity rather than a decrease in recollection-based recognition^44,48^. In line with this previous evidence, our older versus younger participants committed significantly more false alarms. This means they mistook unstudied nouns for studied nouns although the nouns from the distractor list and the new nouns were neither semantically nor phonologically related to the studied nouns. This pattern of recognition results appeared at 30 min and even stronger at one week following learning. The high false alarm rate in older participants at both time points was mostly due to mistaking nouns from the distractor list as nouns from the study list. At the 30-min retrieval time point, older participants made also more false alarms than participants from the middle age group concerning new nouns, which were presented for the first time at this occasion. Hence, older participants committed more false alarm errors due to both interference and poor discrimination. Because the distractor nouns had also been learned initially, namely over one learning run instead of five learning runs, both the target and the distractor nouns were somewhat familiar at the 30-min retrieval and (even more so) at the 1-week retrieval. The pattern of the present and earlier^49,50^ recognition results in older participants suggests that older people dispose of a decreased ability to differentiate between distinct grades of familiarity. Because our participants’ executive functions were not predictive of their false alarm rates, a cognitive monitoring deficit is probably not the cause for the increased rate of false alarms.

A limitation of our study is the uneven subgroup size, especially in the oldest age group, which is owed to data collection during the COVID pandemic, where older more than younger people were forced to stay home. Second, using identical distractors on the recognition test given at the 30-min and the 1-week retrieval rendered the distractors more familiar and therefore harder to discriminate from the targets. Furthermore, the 30-min recognition test allowed for re-encoding and reconsolidation of the target words. Fortunately, this happens at a time point very near to the learning of the target list and before late-phase long-term potentiation and sleep-dependent memory consolidation. Therefore, the 1-week study-test interval was not disturbed by retrieval practice, re-encoding and reconsolidation leaving the memory traces untouched. Third, all words from the learning list were recalled freely at 2 min, 30 min and one week following learning. This procedure bears the advantage of being able to track the fate of individual memories over one week. However, the downside of repeating the recall of all target words is retrieval practice, which may have slowed the forgetting process^51^. We would like to note that retrieval practice might not have clouded an accelerated long-term forgetting in the older participants because a recent study assessed the selective versus complete retrieval of learned items at several time points with the result of an overall diminished forgetting rate with repeated versus selective item retrieval but a comparable forgetting rate in older and younger people over one month^52^.

This study is most relevant for the neuropsychological assessment of older people in the clinical routine. Because increased forgetting over one or more weeks was found in older adults with memory complaints and in patients diagnosed with amnestic mild cognitive impairment^20,21^ and pre-clinical dementia^18,19^, but not in the current examination of healthy older people, accelerated long-term forgetting in older adults might reflect initial subtle dysfunction of the medial temporal lobe in the process of neurodegeneration. Hence, accelerated long-term forgetting in isolation - without initial forgetting over minutes/hours - might mirror an earlier stage of mediotemporal dysfunction than the stage underlying amnestic mild cognitive impairment^21^. We therefore suggest the assessment of delayed memory recall as a method to distinguish between very early pathological and healthy brain aging. In other words, accelerated long-term forgetting might be a biomarker for an incipient neurodegenerative process^5^. To avoid missing accelerated long-term forgetting in the clinical routine, clinical neuropsychologists should include a memory test given several days following learning. For the clinical assessment of accelerated long-term forgetting, we recommend using a process-pure episodic memory test such as a free recall or cued recall test. This is important because aging and dementia concern episodic memory rather than other types of memory such as familiarity and priming. Familiarity and priming might still be preserved in amnestic mild cognitive impairment and they might therefore support the performance on recognition tests^30^ compensating for episodic memory dysfunctions. Hence, familiarity and priming may cloud a problem with episodic memory, when using recognition tests.

Future studies of accelerated long-term forgetting might track memories over even longer periods than one week to capture subtle differences in forgetting rates between age groups, which may escape detection at one week. Future research might also scrutinize the degree to which subtle encoding difficulties provoke an accelerated long-term forgetting^6^ in older people.

## Method

### Participants

We recruited participants through advertisements in local public newspapers and through personal social networks. All participants gave written informed consent to participate in this study. Psychology undergraduates and research assistants applied the cognitive tests either at the participants’ home or at the University Hospital of Bern. Our inclusion criteria for participants were the absence of neurological or psychiatric disorders, no intake of medication with known effects on cognition, normal verbal intelligence as assessed with the multiple choice vocabulary performance test (Mehrfachwahl-Wortschatz-Test^38^), normal executive functions and working memory, and the absence of depression as assessed with a depression screening inventory (DESC^52^). Respecting these inclusion criteria, we enrolled 236 participants (130 women (55%)) at the age of 18–77 years (*M* = 42.26 years, *SD* = 17.16 years). These participants exhibited a mean of 14.27 years of education (*SD* = 2.57 years, range: 7–25 years of education), which is the typical average in the Swiss educational system. We assigned these participants to three age groups analogous to other studies^34^: A young age group (18–29 years), a middle age group (30–59 years), and an old age group (60–77 years). Demographics are listed in Table 1. This study was approved by the Faculty of Humanities’ Ethics Committee at the University of Bern, Switzerland, NR 2018-12-00005. This study was carried out in accordance with the Declaration of Helsinki. Participants were not compensated for their time offered.

### Overview

Each participant took cognitive tests on two days, which were separated by one week. The examination on the first day lasted 90 min and the examination on the second day lasted 15 min. On the first day, participants gave demographic information and took the depression inventory (DESC^52^). Then, they learned 15 unrelated words over five learning runs and later retrieved these words at two minutes and again at 30 min following the last learning run. Finally, they took executive functions tests, a working memory test, and a multiple-choice vocabulary test to approximate their verbal IQ. On the second day, participants took surprise memory tests (recall and recognition), which were not previously announced to prevent participants from rehearsing the words. To prevent rehearsal, we had informed participants that they would fill out further questionnaires on the second day. Indeed, following the unexpected memory tests, participants did fill out a sleep inventory to determine their subjective sleep quality (Pittsburgh Sleep Quality Index, PSQI^53^). We included the assessment of subjective sleep quality because we expected a loss of sleep quality and a related accelerated long-term forgetting due to a poorer sleep-associated memory consolidation for the old age group.

### Material and procedure

We examined the participants’ verbal learning and retrieval performance using a Swiss adaptation of the Rey Auditory Verbal Learning Test (Auditory-Verbal Learning Test (AVLT)^37^. In this test, participants are required to learn fifteen unrelated nouns, which are read to them during each of five consecutive learning runs. Each learning run is immediately followed by a free recall without feed-back given by the experimenter. The five-run-learning is followed by the experimenter’s reading of a distractor word list containing 15 new nouns. These 15 new nouns are neither semantically nor phonologically related to the nouns from the learning list. Participants are required to recall the 15 distractor nouns immediately following their presentation. Next, participants are asked to freely recall the nouns from the initial learning list – this is the so-called ‘2-minute recall’. Thirty minutes and one week following learning, participants are asked again to freely recall the nouns from the initial learning list – these are the so-called 30-minute recall and the 1-week recall.

Both the 30-minute recall and the 1-week recall are followed by a recognition test, which contains the same words at both test time points. The recognition list contains the 15 nouns from the initial learning list, the 15 nouns from the distractor list, and another 15 nouns that were new because they were not presented for learning. The new nouns are not related semantically or phonologically to the other nouns in the recognition list. Hence, for the 30-minute recognition and for the 1-week recognition, we gave participants the same list of 45 nouns to discriminate the target nouns (learning list) from distractor nouns and new nouns.

The dependent variables used for the statistical analyses were verbal learning performance (the sum of all recalled words across the five learning runs), 2-minute recall, 30-minute recall and 1-week recall (number of correctly recalled words), 30-minute recognition and 1-week recognition (total hits (H) and Pr (hits–false alarms) as a discrimination score^54^), absolute recall loss over 1-week (30-min recall – 1-week recall) and absolute recognition loss in terms of H over 1-week (30-min H – 1-week H) as well as absolute recognition loss in terms of Pr over 1-week ((30-min Pr) – (1-week Pr)). Furthermore, we studied the number of false alarms (FA) committed on the recognition test at 30 min and at one week following learning.

Participants’ verbal working memory was assessed with the backward version of the digit span test from the Wechsler Memory Scale Revised^55^; the dependent variable was the largest number of correctly backwardly repeated digits. Executive functions were assessed with the word fluency WF test^56^, which is an adaption of the Controlled Oral Word Association Test (COWAT^57^) given to measure the spontaneous phonemic word production, divergent thinking, and semantic memory retrieval with lexical restrictions (dependent variable: number of produced words), and a Swiss adaptation of the Victoria Stroop Test^58^ to measure cognitive interference control (dependent variable: duration in seconds to name the print color of all words). Because memory consolidation depends on sleep, we had participants fill out the Pittsburgh Sleep Quality Index (PSQI^53^) to reveal their subjective sleep quality. This questionnaire includes questions about sleep duration, sleep latency, sleep quality, sleep disturbances, sleep efficiency (percentage of time spent asleep while in bed), intake of sleep medication, and daytime dysfunction. As dependent variables we used both the total score and all subscale scores. Higher scores mean reduced sleep quality.

### Data analyses

We computed the statistical analyses using the Statistical Package for Social Sciences software for Windows, version 28 (SPSS IBM, NewYork). We used the raw scores for the analysis of verbal memory performance because no normative data for the recall and for the recognition at one week following learning were available at the time.

We first analyzed whether the three age groups differed regarding gender, years of education, depression score, subjective sleep quality, and intelligence (i.e., multiple-choice vocabulary performance). Because the learning curve across the five learning runs and the multiple-choice vocabulary performance were significantly different between the three age groups, we controlled for these two variables when looking at memory retrieval by using the two variables as covariates. Where the assumption of sphericity was violated, we report the Huynh-Feldt corrected degrees of freedom. Although not directly relevant for the hypotheses, we nevertheless explored whether participants’ gender would influence verbal learning performance and would interact with participants’ age when influencing learning by use of ANCOVA (covariate was multiple-choice vocabulary performance) because women usually outperform men on verbal learning in many cultures and the same might be true for Switzerland^59–61^. We also explored whether participants’ gender would influence verbal retrieval performance and interact with age when influencing verbal retrieval performance by use of ANCOVA (controlling for ‘multiple-choice vocabulary performance’ and ‘learning performance’).

To test our hypotheses, we ran the following tests: Repeated measures ANCOVAs with the covariates ‘learning performance’ and ‘multiple choice vocabulary performance’ were run with the independent variables ‘age group’ and ‘time’ (3-levels: 2-min, 30-min, 1-week) and the dependent variables recall performance and recognition performance to test for a main effect of age group and an interaction of age group with time. ANCOVAs with the covariates ‘learning performance’ and ‘multiple choice vocabulary performance’ and the independent variable ‘age group’ were run with the dependent variable ‘recall loss over 1-week’ and the dependent variable ‘recognition loss in terms of H over 1-week’ as well as ‘recognition loss in terms of Pr’ over 1-week to study long-term forgetting more directly. Furthermore, a repeated measures ANCOVA with the covariates ‘learning performance’ and ‘multiple choice vocabulary performance’ was run with the independent variables ‘age group’ and ‘time’ and the dependent variable FA. To scrutinize the nature and origin of the committed false alarms, we divided the FA into the two categories FA to nouns of the distractor list and FA to new nouns. We investigated age group effects on these FA categories for the 30-min and the 1-week recognition test using ANCOVAs with the covariates ‘learning performance’ and ‘multiple choice vocabulary performance’ and ‘age group’ as independent variable. Finally, a multiple linear regression analysis was computed to find out whether demographic variables (age and years of education), intelligence (the individual score of the multiple choice vocabulary performance), learning performance (sum of remembered words across the five runs), subjective sleep quality (the individual scores of all PSQI subscales), working memory, and executive functions (interference control, verbal fluency) would predict verbal forgetting over one week in the entire group of participants. To this end, we computed a multiple linear regression analysis with the delta of recall performance between the 30-minute and the 1-week recall. *R*^*2*^ change was used as an indicator of the individual variance explained by the variables.

## Electronic supplementary material

Below is the link to the electronic supplementary material.

Supplementary Material 1

## Data Availability

The datasets generated during and/or analyzed during the current study are available from the corresponding author on reasonable request.
