# Supplementary material for: Forgetting is comparable between healthy young and old people
Source: Sci Rep. 2024 Dec 28;14:31176. doi: 10.1038/s41598-024-82570-w (PMC11682405; doi:10.1038/s41598-024-82570-w)
Supplement: Supplementary file 1 — Supplementary Material 1 [file 41598_2024_82570_MOESM1_ESM.docx]

**Supplementary Table S1**

*Analysis of false alarms committed to distractor nouns and to new nouns at 30-min recognition and at 1-week recognition*

|  | |  | Age groups |  |  | Test Statistics |  |  | Post-hoc comparisons* |
| --- | --- | --- | --- | --- | --- | --- | --- | --- | --- |
|  | |  | Young age  (18-29 years) | Middle age  (30 – 59 years) | Old age  (60+ years) | Test of significance | *p*-value | Effect size | 1) young age vs. middle age  2) young age vs. old age  3) middle age vs. old age |
|  | 30-min false alarms (distractor nouns), *M (SD)^1^* | | .20 (.53) | .56 (.95) | 1.33 (1.57) | F (2,231) = 6.96 | <.01** | *η_p_ ^2^*=.06 | 1) *ns*  2) *p*<.001, *M* = -.72, 95% CI [-1.12, -.33]  3) *p*<.01, *M* = -.54, 95% CI [-.88, -.21] |
|  | 30-min false alarms (new noun**s)**, *M (SD) ^1^* | | .03 (.18) | .01 (.10) | .18 (.58) | F (2,231) = 3.28 | <.05* | *η_p_ ^2^*=.03 | 1) *ns*  2) *ns*  3) *p*<.05, *M* = -.11, 95% CI [-.22, -.01] |
|  | 1-week false alarms (distractor nouns), *M (SD) ^1^* | | .64 (1.10) | 1.30 (1.74) | 2.71 (2.81) | F(2,231) = 8.00 | <.001*** | *η_p_ ^2^*=.07 | 1) *ns*  2) *p*<.001, *M* = -1.47, 95% CI [-2.21, -.73]  3) *p*<.01, *M* = -1.07, 95% CI [-1.70, -.43] |
|  | 1-week false alarms (new noun**s)**, *M (SD) ^1^* | | .10 (.34) | .46 (.96) | .80 (1.38) | F(2,231) = 1.52 | .22 |  |  |

*Note*. *M*: mean; *ns*: not significant, *SD*: standard deviation. Significance value: **p*<.05, ***p*<.01 ****p*<.001. ^1^controlled for learning and multiple choice vocabulary performance.
